# A Self-Calibration Stitching Method for Pitch Deviation Evaluation of a Long-Range Linear Scale by Using a Fizeau Interferometer

**DOI:** 10.3390/s21217412

**Published:** 2021-11-08

**Authors:** Xin Xiong, Yuki Shimizu, Hiraku Matsukuma, Wei Gao

**Affiliations:** 1 Department of Finemechanics, Tohoku University, Sendai 980-8579, Japan; xiongxin@zju.edu.cn (X.X.); hiraku.matsukuma.d3@tohoku.ac.jp (H.M.); i.ko.c2@tohoku.ac.jp (W.G.); 2 The State Key Lab of Fluid Power and Mechatronic Systems, Zhejiang University, Hangzhou 310027, China; 3 Division of Mechanical and Space Engineering, Graduate School of Engineering, Hokkaido University, Kita 13, Nishi 8, Kita-ku, Sapporo 060-8628, Japan

**Keywords:** optical encoder, linear scale, pitch deviation, stitching interferometry, self-calibration

## Abstract

An interferometric self-calibration method for the evaluation of the pitch deviation of scale grating has been extended to evaluate the pitch deviation of the long-range type linear scale by utilizing the stitching interferometry technique. Following the previous work, in which the interferometric self-calibration method was proposed to assess the pitch deviation of the scale grating by combing the first-order diffracted beams from the grating, a stitching calibration method is proposed to enlarge the measurement range. Theoretical analysis is performed to realize the *X*-directional pitch deviation calibration of the long-range linear scale while reducing the second-order accumulation effect by canceling the influence of the reference flat error in the sub-apertures’ measurements. In this paper, the stitching interferometry theory is briefly reviewed, and theoretical equations of the *X*-directional pitch deviation stitching are derived for evaluation of the pitch deviation of the long-range linear scale. Followed by the simulation verification, some experiments with a linear scale of 105 mm length from a commercial interferential scanning-type optical encoder are conducted to verify the feasibility of the self-calibration stitching method for the calibration of the *X*-directional pitch deviation of the linear scale over its whole area.

## 1. Introduction

Due to their low cost, high resolution, and robustness, optical linear encoders are widely employed for precision positioning applications, such as semiconductor manufacturing, precision machine tools, and coordinate measuring machines (CMMs) [1,2,3,4]. Among the optical encoders, the interferential scanning-type optical encoder with a high precision scale grating has the highest performance [5,6]. The optical setup of the optical linear encoder is arranged to produce interference signals generated by combining the positive and negative first-order diffracted beams from the linear scale [7]. The displacement of the linear scale can then be obtained by analyzing the monitored interference signals by using the interpolation technique. Since the period of the interference signal is determined by the pitch of the linear scale, the measurement accuracy of the linear encoder will be directly influenced by the pitch deviation of the linear scale [8]. Meanwhile, the spanning width of the linear scale directly determines the measurement range of the linear encoder. Scale grating that has a length longer than 100 mm is required for long-range precision positioning [7,9,10]. The interest in expanding the evaluation area of the linear scale is growing, and it also increases the difficulty to calibrate the pitch deviation of the long-range type linear scale along its whole length.

The line scale comparator is used as the standard solution to accurately evaluate the pitch deviation of the one-axis linear scale used in the linear encoder [11,12]. However, it is burdensome to construct such a system in a research lab with a limited budget. On the other hand, although the scanning probe microscopes (SPMs) can be applied to provide an image of a small part of the linear scale [13,14,15], it is limited by the slow scanning speed and small scanning range. These limitations would hinder the efficient evaluation of the pitch deviation of the long-range linear scale over its whole area. Meanwhile, the measured pitch maps using SPMs need to be further processed for the evaluation of the pitch deviation at each position. In responding to the above issues, an interferometric calibration method has been proposed to evaluate the pitch deviation of the scale grating using a Fizeau form interferometer through wavefront analysis [16]. The proposed method is further improved to a self-calibration method so that the influence of the reference flat error in the Fizeau interferometer can be removed [17,18,19].

However, according to the measurement principle of the form interferometer, the measurement range is limited by the size of its reference optical flat [20]. Using a larger size interferometer so that the beam of the interferometer can be expanded to completely cover the long-range linear scale could be a possible solution [21]. Nonetheless, it suffers from defects such as being slow to reach thermal equilibrium and sometimes it is impossible to prevent long propagation of distance, which would result in thermal fluctuations and distortion in higher spatial frequencies [22]. In addition, the cost of acquisition and setup can be high for such a large system. On the other hand, the stitching interferometry technique has long been used to evaluate the *Z*-directional surface form error of the large size optical components [23,24,25,26]. Nevertheless, the stitching calibration of *X*-directional pitch deviation of large size scale grating has not been conducted yet. Meanwhile, with the reference flat error that exists in each sub-aperture, an accumulative second surface error could be generated by the conventional stitching algorithm [27,28], which would result in unwanted power and astigmatism terms. Since calibration of the reference flat would cost time and require other precision optical components, the self-calibration technique is needed to remove the systematic error.

In this paper, a Fizeau form interferometer is used to evaluate the *X*-directional pitch deviation of a reflective-type long-range linear scale with a self-calibration stitching method. With the proposed method, the *X*-directional pitch deviation of a long-range linear scale over its whole area can be self-calibrated in a short time with high throughput. At first, the basic stitching interferometry technique is briefly introduced. The self-calibrated stitching method for the long-range type linear scale pitch deviation evaluation is then proposed by considering the additional phase shift errors introduced in the stitching measurement, which is then verified through a simulation. Finally, experiments are conducted with a long-range-type linear scale used in a commercial optical linear encoder.

## 2. Principle

### 2.1. Basic Principle of Z-Directional Surface Form Stitching Interferometry

**Figure 1:**
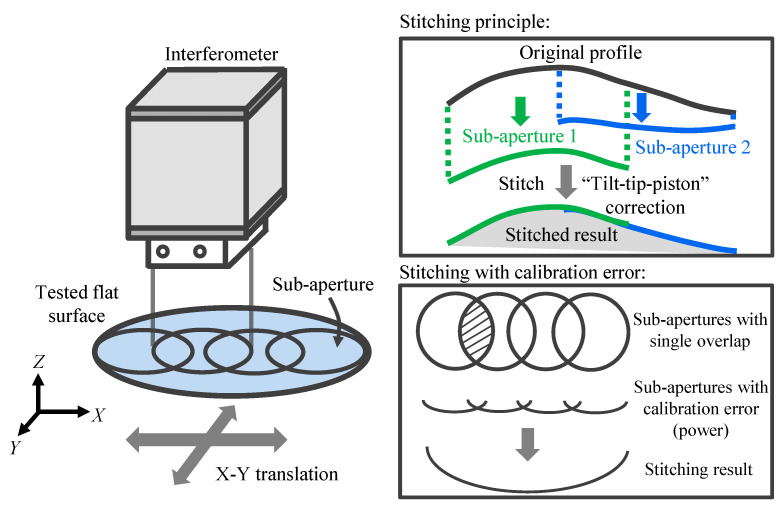
Schematic of the concept of the *Z*-directional surface form stitching interferometry and the influence of calibration error on stitching result.

The basic idea of stitching interferometry is quite simple. If an optical component is too large to be measured, the measurement area can be separated into several small overlapped pieces (sub-apertures) and stitched together so that the surface form of the large optical component can be evaluated by using a standard “small” interferometer [29,30]. Figure 1 shows the schematic of the concept of stitching interferometry, which indicates that the stitching technique can enable the measurement of the large size optical component by using a “small size” interferometer. The final result is obtained by taking multiple overlapping images of the large component, and numerically “stitching” these sub-apertures together by computing a correcting “Tip-Tilt-Piston” correction for each sub-aperture [27,31]. In addition, for some special applications such as stitching the optical component with a large convex aspheric surface or a high numerical aperture cylindrical optics, other additional corrections except the “Tip-Tilt-Piston” can also be conducted in the stitching process by using a well-designed algorithm [32,33]. According to the principle of stitching interferometry, it would be helpful to enlarge the measurement area and improve the lateral resolution with little investment in the additional devices (usually a precision motion stage, which is available in most of the optical lab). However, for the case of the stitching calibration of long-range linear scale, the sub-aperture topography is one-dimensional, and the calibration error will propagate fully and could result in a second-order accumulative error when applying the stitching algorithm. Figure 1 shows the effect of the calibration error on the final stitching result. From the Figure, it can be deduced that as the measured object size increases, the accumulative second-order stitching error would be enlarged correspondingly and should not be neglected in the stitching calibration.

### 2.2. Self-Calibrated Stitching of X-Directional Pitch Deviation of the Long-Range Linear Scale

**Figure 2:**
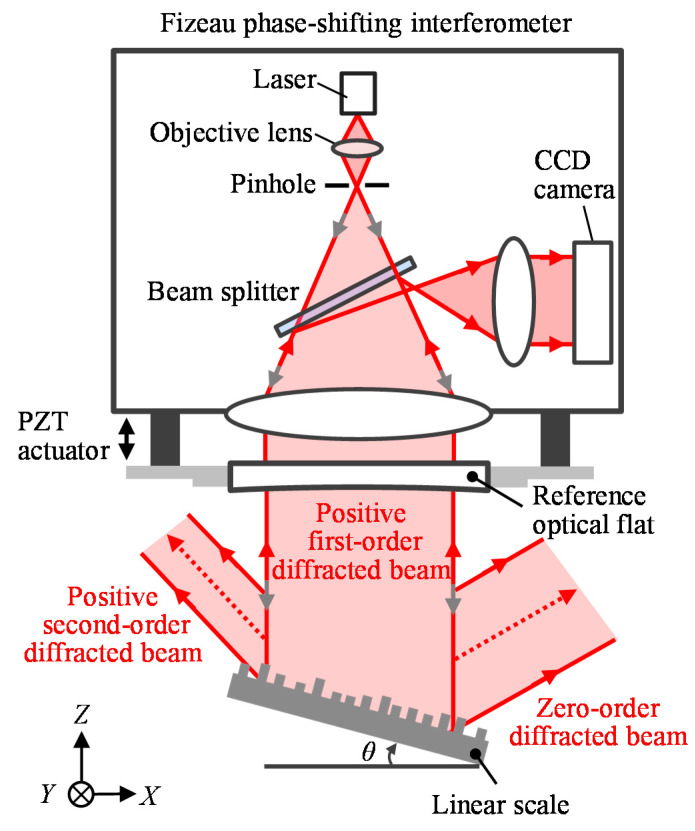
Measurement of the positive first-order diffracted beams from the grating by using the Littrow setup.

The stitching calibration method for the long-range linear scale pitch deviation evaluation can be developed by integrating the stitching interferometry for the *Z*-direction phase error compensation, just as it is used to evaluate the surface form of a large size optical component. On the other hand, since the periodic pattern of the linear scale is arranged along the *X*-direction, the *X*-directional stitching for pitch deviation should also be included. The pitch deviation evaluation of the linear scale requires the measurement of the first-order diffracted beams from the linear scale by setting it in the Littrow setup as Figure 2 shows. In the setup, the diffracted beam can be back-reflected directly to the direction of the incident beam and be captured by the interferometer. When using the Fizeau interferometer for the measurement of the diffracted wavefront of the linear scale, the positive and negative first-order phase outputs of the interferometer can be expressed by [16]

(1) $$I_{X+1}(x,y)=2\pi \frac{e_{X}(x,y)}{g}+2\pi \frac{2e_{Z}(x,y)}{\lambda }\cos\theta -2\pi \frac{2e_{R}(x,y)}{\lambda }$$

(2) $$I_{X-1}(x,y)=-2\pi \frac{e_{X}(x,y)}{g}+2\pi \frac{2e_{Z}(x,y)}{\lambda }\cos\theta -2\pi \frac{2e_{R}(x,y)}{\lambda }$$

where *I_X_*_+1_(*x*, *y*) and *I_X_*_−1_(*x*, *y*) are the positive and negative first-order phase outputs, respectively. *e_Z_*(*x*, *y*) and *e_R_*(*x*, *y*) are *Z*-directional out-of-flatness of the scale grating and reference flat. *λ* is the wavelength of the light source of the Fizeau interferometer, *e_X_*(*x*, *y*) is the *X*-directional pitch deviation of the linear scale, *g* is the nominal pitch of the grating, *θ* is the Littrow angle. It should be pointed out that in the experiment only the first-order diffracted beams from the grating are utilized for calibration. Although higher-order diffracted beams can also be used to calibrate pitch deviation, the measurement could suffer from low diffraction efficiency and loss of lateral information when applying a larger tilt angle. Consequently, the *X*-directional pitch deviation of the linear scale can be calculated as [16]

(3) $$e_{X}(x,y)=\frac{g}{4\pi }[I_{X+1}(x,y)-I_{X-1}(x,y)]$$

**Figure 3:**
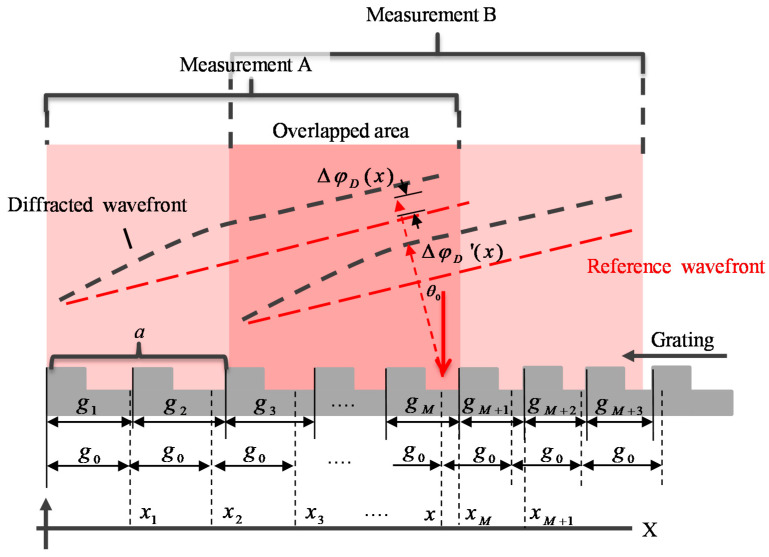
Schematic of the phase error generated by the *X*-directional pitch deviation in stitching measurement.

In the stitching measurement, multiple measurements are required by shifting the linear scale along the *X*-direction to obtain several overlapped results and stitching them together. When measuring after shifting the linear scale with a known distance, phase error would be generated by the pitch deviation in different areas. According to the phase shift theory of diffraction grating, the phase error caused by the pitch deviation of the scale grating is accumulated and can be stitched. Figure 3 shows the case that two adjacent areas *A* and *B* with an overlapped area of the scale grating are measured by the Fizeau interferometer. The translation distance is a known value *a*. In the figure, *g*_0_ represents the nominal pitch, *g_i_* (*i* = 1, 2, 3, …, *M*) represents the actual pitch. More conveniently, the pitch at any coordinate can be expressed as the pitch function *g*(*x*). When the linear scale is measured at area *A*, the phase error caused by the pitch deviation at coordinate *x* can be calculated as

(4) $$\Delta \phi_{D}(x)≅\frac{2m\pi }{g_{0}}\int_{0}^{x}\frac{\left(g_{0}-g(x)\right)}{g(x)}dx=\frac{2m\pi }{g_{0}}\int_{0}^{x}\frac{\Delta g(x)}{g(x)}dx$$

After moving the linear scale to the next area *B* with a distance of value *a*, the phase error caused by the pitch deviation at the coordinate *x* would be changed to

(5) $$\Delta \phi {’}_{D}(x)≅\frac{2m\pi }{g_{0}}\int_{a}^{x}\frac{\left(g_{0}-g(x)\right)}{g(x)}dx=\frac{2m\pi }{g_{0}}\int_{a}^{x}\frac{\Delta g(x)}{g(x)}dx$$

Therefore, at the same position *x* from the field-of-view (FOV) of the interferometer, the phase error is generated due to the change of the evaluated area. The phase difference caused by the shifting process can be expressed as

(6) $$\Delta \varphi (x)=\Delta \phi_{D}(x)-\Delta \phi {’}_{D}(x)=\frac{2m\pi }{g_{0}}\int_{0}^{a}\frac{\left(g_{0}-g(x)\right)}{g(x)}dx=c$$

Equation (6) indicates that the difference of the phase shift caused by the two measurements is only a constant value *c*, and the additional piston error caused by the pitch deviation should also be corrected. On the other hand, regarding the grating itself as a rigid body in the stitching measurement, the piston/tilts errors would be generated in the shifting process. Combing the additional phase errors caused by the *X*-directional pitch deviation and *Z*-directional out-of-flatness error in the stitching measurement, the first-order phase outputs in the overlapped area can be expressed as

(7) $$I_{X\pm 1,i}(x,y)=\pm 2\pi \frac{e_{X,i}(x,y)}{g}+\frac{4\pi }{\lambda }[e_{Z,i}(x,y)\cos\theta -e_{R,i}(x,y)]+a_{X\pm 1,i}x+b_{X\pm 1,i}y+c_{X\pm 1,i}$$

where the coefficients *a_X_*_±1*,i*_, *b_X_*_±1*,i*_, are the tilt coefficients along the *X*- and *Y*- directions in *i*-th sub-aperture corresponding to the positive and negative first-order diffracted beams measurement, respectively, while *c_X_*_±1*,i*_ is the coefficient of the piston error along the *Z*-direction in *i*-th sub-aperture of the positive and negative first-order diffracted beams measurement. To correct the tilts and piston error and obtain the full aperture of the phase maps, it is desired to minimize the sum of the square differences for all overlapped areas at the same time [32]

(8) $$\min=\sum_{i=1\ldots N}\sum_{j=1\ldots N(j\neq i)}^{j\cap i}{\left[(I_{k,i}(x,y)+a_{k,i}x+b_{k,i}y+c_{k,i})-(I_{k,j}(x,y)+a_{k,j}x+b_{k,j}y+c_{k,j})\right]}^{2}$$

where *N* represents the total number of the sub-apertures, *k* = *X* ± 1, *i* and *j* represent different *i*-th/*j*-th sub-aperture. The error coefficients *a_k_,_i/j_*, *b_k_,_i/j_*, and *c_k_,_i/j_* can be calculated by solving Equation (8) with least-square techniques [34]. The equation is differentiated and the error coefficients can be calculated by solving a linear matrix equation. Assuming the overlap area is square (*n* × *n* pixels), the time-complexity of the optimization of the equation would be proportional to *n*^2^*N*. Equation (8) can be applied to calculate the error coefficients for each sub-aperture in the two diffraction orders. It is noted that the *Z*-directional rotational error is ignored due to the small effect of the cosine error. In addition, the *X*-directional displacement error is also not considered since the resolution of the precision stage used to translate the measured optics is usually better than that of the form interferometer. According to Equation (3), by using the stitched positive and negative first-order phase outputs, the pitch deviation of the long-range linear scale over its whole area can be evaluated. Since the reference flat error component contained in the *i*-th positive and negative first-order diffracted beams are the same. Therefore, the second-order error component caused by the reference flat error component would also be the same in the final stitched phase maps, which can be wiped out by carrying out the differential operation. Based on the self-calibration principle, a more direct approach is to stitch the pitch deviation maps in each sub-aperture together by minimizing the following function

(9) $$\min=\sum_{i=1\ldots N}\sum_{j=1\ldots N(j\neq i)}^{j\cap i}{\left[(e_{X,i}(x,y)+a_{k,i}x+b_{k,i}y+c_{k,i})-(e_{X,j}(x,y)+a_{k,j}x+b_{k,j}y+c_{k,j})\right]}^{2}$$

where *e_X,i_*(*x*, *y*) is the pitch deviation in *i*-th aperture, which is calculated by

(10) $$e_{X,i}(x,y)=\frac{g}{4\pi }[I_{X+1,i}(x,y)-I_{X-1,i}(x,y)]$$

**Figure 4:**
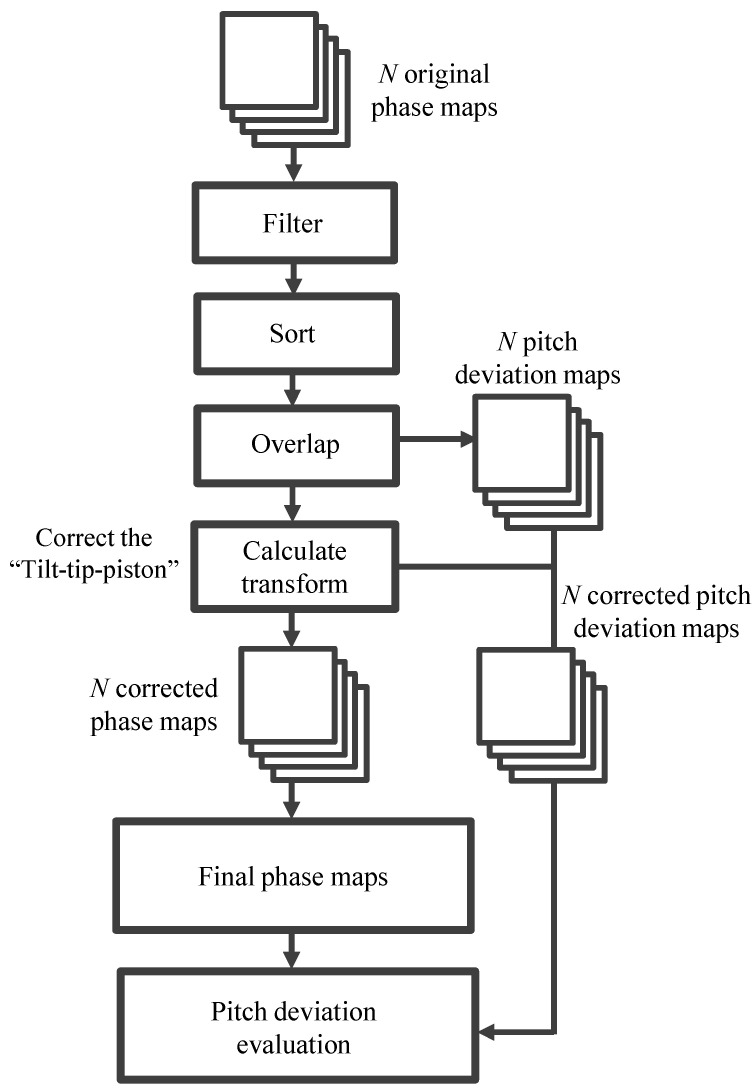
Flow diagram indicating the working principle and the data flow of the stitching algorithm for the calibration of the long-range linear scale pitch deviation.

From the above analysis, the procedure of the interferometric self-calibrated stitching of the long-range linear scale pitch deviation can thus be summarized, as shown in Figure 4. In the stitching measurement, *n* phase maps are first collected continuously by moving the tested object or the interferometer. Since one-dimensional stitching is conducted for the linear scale, the arrangement of the position of each sub-aperture should be carefully designed to ensure there is enough overlap area between adjacent sub-apertures. With the existence of the calibration errors and dynamic errors (i.e., thermal, mechanical, etc.), the stitching accuracy could be influenced if only a single-overlap strategy with a small overlap area between adjacent sub-apertures is performed. Although there is no rule-of-thumb of the selection of the best overlap ratio, a double-overlap strategy is preferred so that each overlap could be constrained by an independent sub-aperture and the stitching error can be reduced. The digital filtering method is then used to remove the high-frequency noise components in the phase maps and enhance the performance of the stitching algorithm. The next step is to locate the unified global phase map center and sort the different images according to the moving distance between each phase map. With the arrangement of each image, the overlapped area of each image in the global coordinate can thus be determined. Before applying the stitching algorithm, the reference image should be decided. Usually, the image closest to the global image center could act as the global reference and will remain fixed throughout the whole stitching process. Then the objective is to find a transformation that can describe the misalignment of the sub-image sets with respect to the global reference image and then correct for the misalignment. The *n* phase maps can, thus, be stitched together to obtain the final phase maps. Finally, the pitch deviation can be calibrated using Equation (3). On the other hand, as demonstrated in the previous analysis, another approach to obtain the pitch deviation of the scale grating is to directly stitch the pitch deviation in each sub-aperture together by using Equations (9) and (10).

## 3. Simulations

Following the theoretical analysis described in the previous sections, numerical calculations are conducted to verify the proposed algorithm. In the simulation, the nominal pitch of the scale grating is set to be 1.6 μm, while the wavelength of the laser source is set as 632.8 nm. First, the form errors of the scale grating *e_Z_*(*x*, *y*) and *e_X_*(*x*, *y*), as well as the reference flat error *e_R_*(*x*, *y*), are simulated with given functions. Note that in the simulation, the *X*- and *Y*- coordinate of the linear scale is normalized to [−1, 1] for the sake of simplicity. Then, the phase outputs *I_X_*_±1_(*x*) are simulated by using the previously given form errors of the scale grating and reference flat. Next, the simulated phase outputs are separated into several sub-apertures with known overlap information. Finally, the pitch deviation of the scale grating is reconstructed with the proposed self-calibration method by using both the stitched phase outputs or directly stitching the pitch deviation in each sub-aperture together.

As the first step of the numerical calculation, each of the form errors of the scale grating and the reference flat is given as follows

$$\begin{array}{ll} e_{Z}(x,y) & =0.31-26.1x+3.3y+147.9x^{2}+18.1y^{2}+20.7x^{3}-0.53x^{2}y+2.76xy^{2}-2.9xy^{2}\\ & -154.7x^{4}+12.1x^{3}y+16.1x^{2}y^{2}+12.1xy^{3}-27.5y^{4} \end{array}$$

$$\begin{array}{ll} e_{R}(x,y) & =-8.1-1.1x-4.3y-0.066x^{2}-22.5y^{2}+0.14x^{3}+0.44x^{2}y-3.63xy^{2}-45x^{4}\\ & -0.55x^{3}y+19.1x^{2}y^{2}+1.02xy^{3}-1.02y^{4} \end{array}$$

$$\begin{array}{ll} e_{X}(x,y) & =-0.84+188.7x-4.2y-93.4x^{2}-1.8xy-12.8y^{2}-183.8x^{3}+0.76x^{2}y+2.29xy^{2}\\ & -61x^{4}-1.2x^{3}y+7.47x^{2}y^{2}+2.8xy^{3}+11y^{4} \end{array}$$

**Figure 5:**
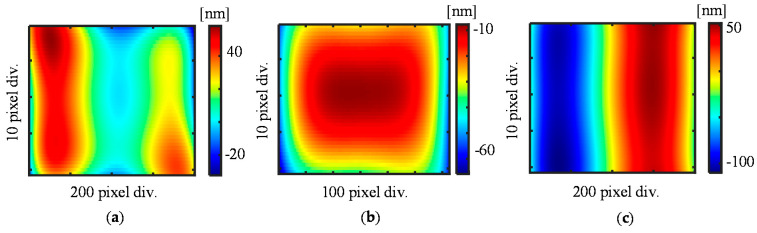
Simulation results of the form errors of a linear scale. (**a**) Out-of-flatness error; (**b**) Reference flat error; (**c**) *X*-directional pitch deviation.

**Figure 6:**
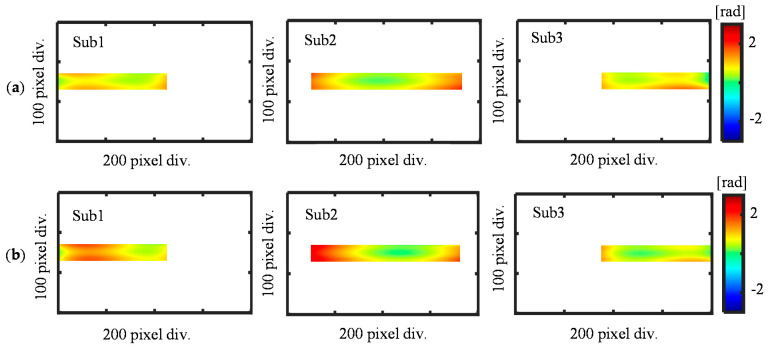
Simulation results of the positive and negative first-order diffracted beams. (**a**) Sub-apertures of the positive first-order diffracted beams; (**b**) Sub-apertures of the negative first-order diffracted beams.

Figure 5 shows the simulated results of the form errors. After the form errors are simulated, the phase outputs I*_X_*_±1_(x) are then calculated according to Equations (1) and (2). To simplify the analysis, three sub-apertures with a rectangular shape are applied totally to separate the simulated phase outputs I*_X_*_±1_(x) with known overlap information, as shown in Figure 6.

**Figure 7:**
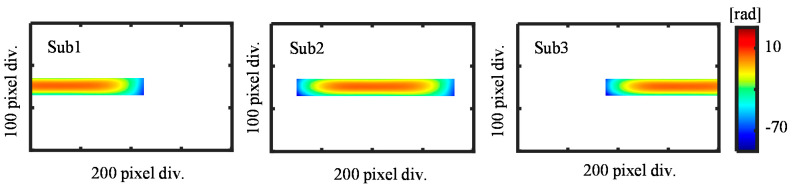
Simulated reference flat error for each sub-aperture.

The reference flat error is also considered and added to the phase output in each sub-aperture, as shown in Figure 7. To simulate the possible phase errors caused by the tilt, tip, and piston, the values of the error coefficients a, b, and c are randomly generated for sub-aperture 1 and 3, while these error coefficients are set to be zero for sub-aperture 2 since it is arranged as the reference sub-aperture.

**Figure 8:**
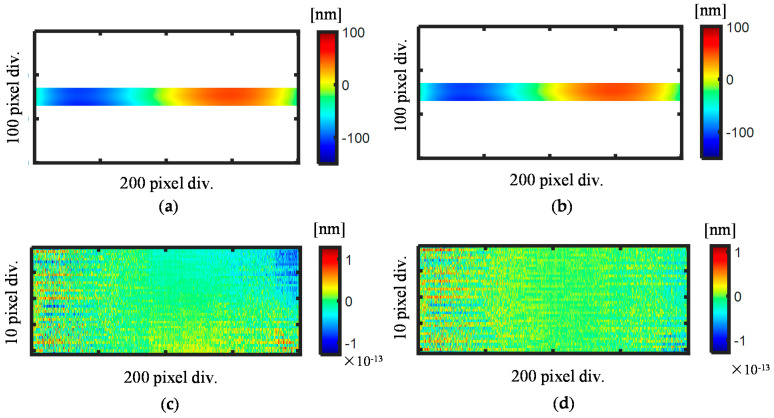
Reconstructed pitch deviation results using the stitched first-order diffracted beams and the pitch deviation maps. (**a**) Evaluated pitch deviation obtained by using the stitched first-order diffracted beams; (**b**) Evaluated pitch deviation obtained by directly stitching the pitch deviation maps; (**c**) Difference obtained from the two results (**a**,**b**); (**d**) Difference between the evaluated pitch deviation and the simulated pitch deviation in Figure 5c.

Figure 8 shows the reconstructed pitch deviation using the proposed self-calibration method. The pitch deviation of the scale evaluated by using the stitched phase outputs I*_X_*_±1_(x) is shown in Figure 8a and the evaluation result obtained by directly stitching the pitch deviation is shown in Figure 8b. Figure 8c shows the difference between the two reconstruction results, which indicates that the two reconstruction results are almost the same with a small difference on the level of 10–13 nm. Figure 8d presents the differential result between the reconstructed pitch deviation and simulated pitch deviation. The results verify that the self-calibration stitching method has a stitching accuracy at the level of 10–13 nm.

## 4. Experiments

### 4.1. Experimental Setup

**Figure 9:**
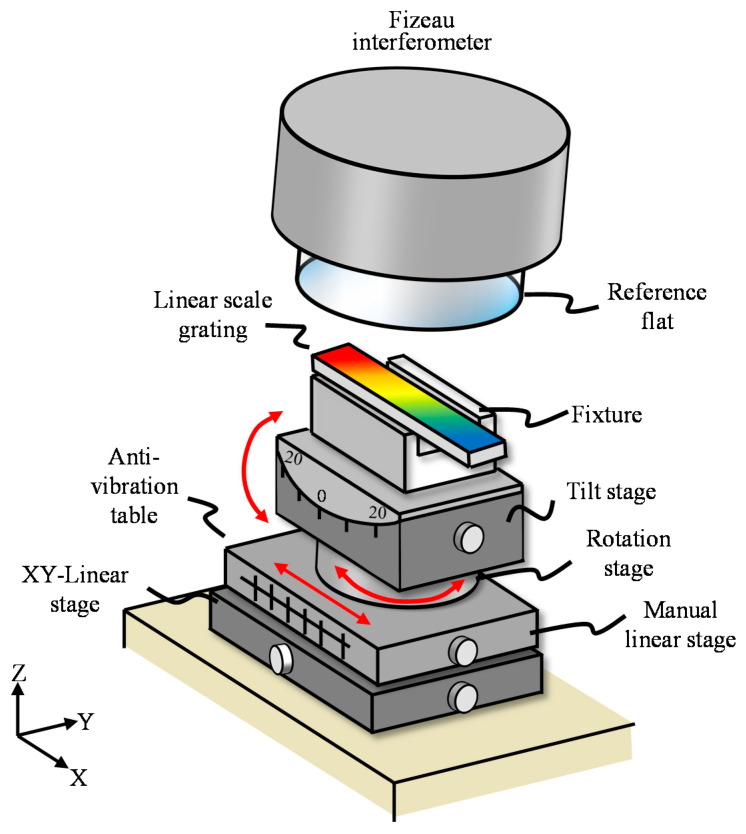
Experimental setup with a commercial Fizeau interferometer.

A commercial Fizeau interferometer (Verifire^TM^, Zygo Corp., Middlefield, CT, USA), which has a measurement range of 102 mm in diameter, was used in the experiment. The wavelength of the light source was 632.8 nm. The resolution and accuracy along the *z*-axis were 0.05 nm and *λ*/20, respectively. Figure 9 shows the experiment setup with major components. A precision tilt stage (TS-211, Chuo Precision Industrial Co., Ltd., Tokyo, Japan) was employed in the experiments to set the linear scale in the Littrow configuration. A precision two-axis positioning stage with a resolution of 10 μm and a manual rotary stage was employed to adjust the in-plane position of the linear scale. In addition, a precision manual linear stage with a resolution of 100 μm and a travel range of 100 mm was used. It should be noted that the in-plane position of the linear scale could easily be determined by locating the edges of the linear scale to coincide with those of the CCD image from the visual feedback system. Meanwhile, highly precise in-plane positioning of the linear scale was not required since the lateral resolution of the CCD camera was larger than that of the positioning stages employed in the experiments.

In the experiment, the linear scale having a nominal pitch of 1.6 μm over an area of 5 mm × 105 mm was employed as the measurement specimen. The linear scale was measured through three shots for each diffraction order and the diffracted beams in different sub-apertures were measured by reciprocating the linear stage. First, the positive first-order diffracted beam of the right part of the linear scale was measured by tilting the linear scale clockwise. Then, the scale grating was translated forward with a distance of 12 mm to measure the positive first-order diffracted beam from the middle part of the linear scale. At last, the positive first-order diffracted beam from the left part of the linear scale was measured by moving the scale slightly forward with a distance of 13 mm. Once the measurements of the positive first-order diffracted beams in each sub-aperture were finished, the linear scale was moved back to the initial position. The linear scale was then tilted counter-clockwise to measure the negative first-order diffracted beams. The measurement procedure of the negative first-order diffracted beams in each sub-aperture was the same as the measurement of the positive first-order diffracted beams. In each measurement, the number of the observed interference fringes in the visual feedback system was reduced to a minimum through adjusting the manual tilt stage to reduce the influence of the angular misalignment from the Littrow angle. In addition, to reduce the influence of environmental noise, three repetitive measurements were conducted in each position. Excluding the warm-up and pre-adjustment time, it took approximately 20 min to conduct all the measurements including the translation of the linear stage and the tilt operation of the linear scale grating. The measured phase outputs were then processed offline for the analysis with the self-calibration stitching method.

### 4.2. Experiment Results and Discussions

**Figure 10:**
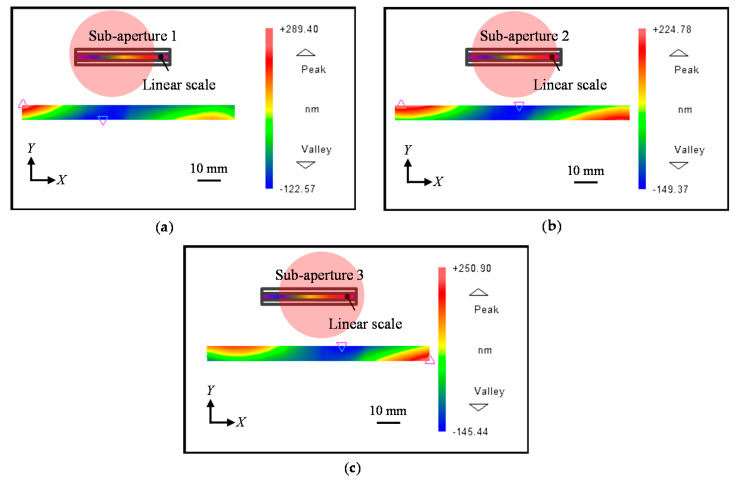
Measured positive first-order diffracted wavefront from the linear scale grating. (**a**) *X*-directional positive first-order diffracted beam from Sub-aperture 1; (**b**) *X*-directional positive first-order diffracted beam from Sub-aperture 2; (**c**) *X*-directional positive first-order diffracted beam from Sub-aperture 3.

**Figure 11:**
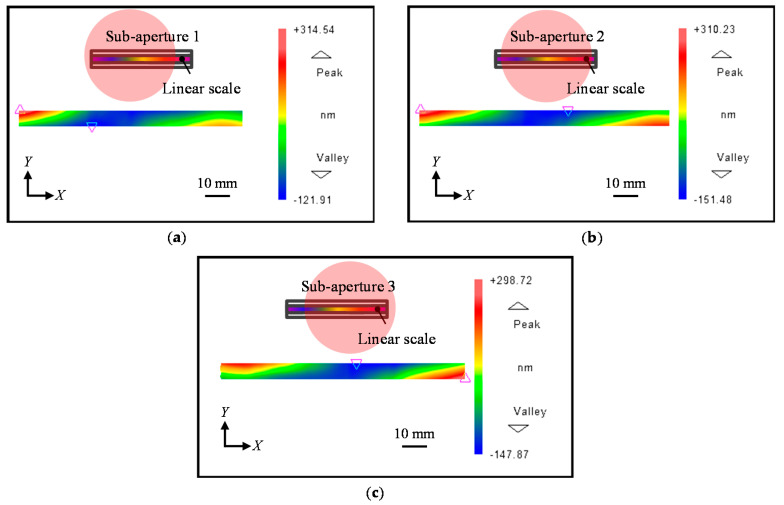
Measured negative first-order diffracted wavefront from the linear scale grating. (**a**) *X*-directional negative first-order diffracted beam from Sub-aperture 1; (**b**) *X*-directional negative first-order diffracted beam from Sub-aperture 2; (**c**) *X*-directional negative first-order diffracted beam from Sub-aperture 3.

Figure 10 shows the observed positive first-order diffracted beams from each sub-aperture, while Figure 11 shows the measured negative first-order diffracted beams from sub-aperture 1 to sub-aperture 3, respectively.

Sub-aperture 2 was arranged as the reference aperture since it has the maximum overlap area with the other two sub-apertures. The error coefficients (*a*, *b*, *c*) of sub-aperture two were then calculated using Equations (8) and (9) for different stitching strategies. The calculated error coefficients of each sub-aperture are summarized in Table 1.

**Figure 12:**
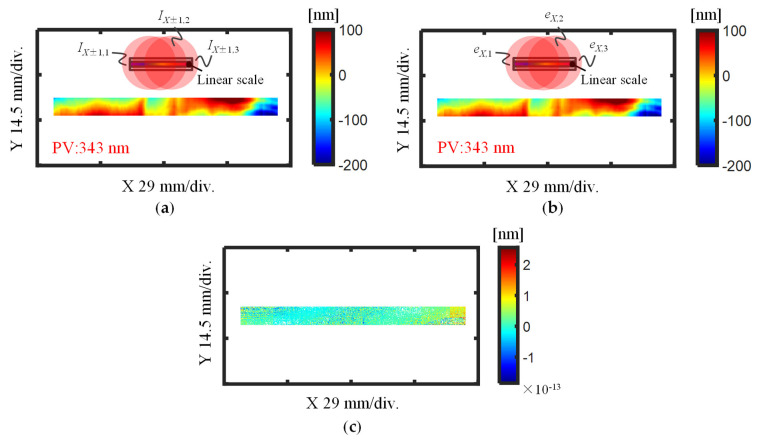
Evaluated *X*-directional pitch deviation of the linear scale through different stitching strategies. (**a**) Pitched deviation evaluated from stitched first-order diffracted beams; (**b**) Pitched deviation evaluated by stitching the pitch deviation from each sub-aperture; (**c**) Difference obtained from the two results in (**a**,**b**).

Figure 12 shows the pitch deviation results obtained in the previously described two different strategies. The pitch deviation calculated from the stitched phase maps is shown in Figure 12a, which indicates that the pitch deviation over the whole area of the linear scale had a peak-to-valley (PV) value of 343 nm. Meanwhile, Figure 12b shows the pitch deviation of the linear scale calculated by stitching the pitch deviation in each sub-aperture, which also had a PV value of 343 nm. Figure 12c shows the difference between the 2D pitch deviation maps obtained by the two different methods. From the small differential result, it is noted that almost the same results were obtained by using the two different self-calibration stitching methods.

**Figure 13:**
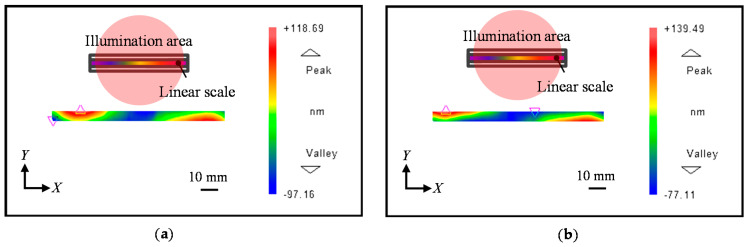
Measured first-order diffracted beams from the linear scale with one-shot measurement. (**a**) *X*-directional positive first-order diffracted beam; (**b**) *X*-directional negative first-order diffracted beam.

**Figure 14:**
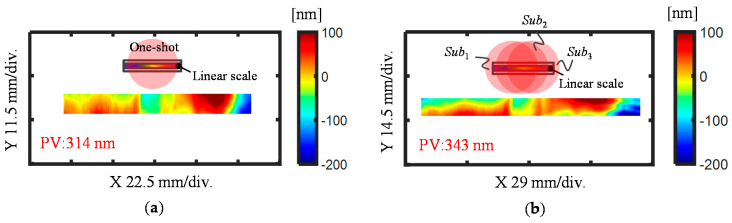
Evaluated pitch deviation of the linear scale through one-shot and three-shot measurements. (**a**) Pitch deviation of the linear scale evaluated with a one-shot measurement; (**b**) Pitch deviation of the linear scale evaluated with a three-shot measurement and stitching method.

To verify the stitching calibration results, a one-shot experiment was also conducted. By zooming out the observation area of the CCD camera, most of the scale area (about 5 mm × 101 mm) was covered by the illumination area of the one-shot measurement. The positive and negative first-order diffracted beams of the linear scale were evaluated by tilting the linear scale clockwise and counter-clockwise, as shown in Figure 13a,b, respectively. Figure 14 presents the calculated pitch deviation of the linear scale by using the measured wavefronts from the one-shot measurement and the results obtained by using the self-calibrated stitching method. Figure 14a shows the measured pitch deviation with the one-shot measurement had a PV value of 314 nm, which is slightly smaller than that of the self-calibrated stitching result. Nevertheless, the high correspondence of the topography features in the two 2D pitch deviation maps verified the feasibility of the proposed method for pitch deviation stitching calibration.

**Figure 15:**
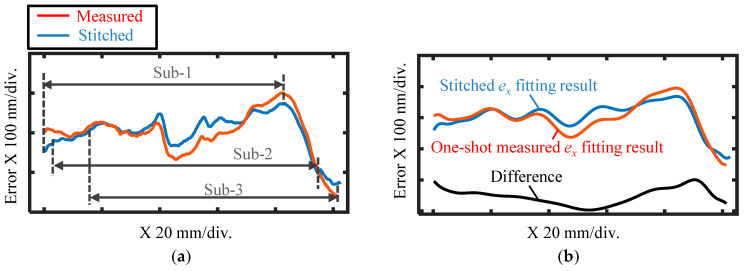
Comparison of the *X*-directional averaged cross-sections of the measured and stitched pitch deviation through one-shot and three-shot measurements. (**a**) *X*-directional averaged cross-section of the measured and stitched pitch deviation results; (**b**) Reconstruction results of the two averaged pitch deviation cross-sections and their difference.

To further verify the detail, the averaged *X*-direction cross-section of the two results was calculated and compared with each other. Figure 15a shows the comparison of the averaged *X*-directional cross-sections of the two calibration results in Figure 14. The pitch deviation was then reconstructed by considering the cosine value of the Littrow angle with a 20-order polynomial function using the least-square technique [17]. The difference was then calculated with the two reconstruction results, as shown in Figure 15b. From the figures, the two averaged cross-sections show good correspondence with each other, and the difference was within the range of ±50 nm over the whole calibration area, verifying the capability of the proposed self-calibration stitching method for long-range linear scale pitch deviation evaluation.

The pitch deviation of a long-range linear scale was successfully calibrated using the proposed stitching calibration method. The same results are obtained by using different stitching strategies with the self-calibration method. Considering the lengths of the industrial used commercial linear scale are mostly within the range of 300 mm [9], these could be measured with a commercial interferometer with three to four shots. The measurement results obtained using the proposed self-calibrated stitching method are thus representative, indicating the feasibility of the proposed method to measure a 200 mm or 300 mm long linear scale. On the other hand, it was found that there was a difference between the stitched pitch deviation with three-shot measurement and the evaluated pitch deviation with one-shot measurement. The difference could be mainly caused by the *X*-directional straightness error of the linear stage, which should be calibrated before the stitching measurement. The positioning error related to the straightness error and the linear stage could also influence the stitching accuracy, although it is usually ignored since the lateral resolution of the interferometer is worse than that of the precision positioning stage [35,36]. It should be pointed out that systematic errors such as the calibration error of the phase shifter as well as the possible speckle effect could also influence the measurement results and reduce the stitching accuracy. Well-designed PSI algorithms and speckle reduction methods could be applied to address these issues [37,38]. Other uncertainties such as the environmental noise and the uncertainty related to the interferometer are estimated to be several nanometers and are not considered in the analysis [19].

It should be noted that the main focus of this paper is to verify the feasibility of applying the proposed self-calibration stitching method for the long-range linear scale pitch deviation calibration. The proposed method could be extended to calibrate the *Z*-directional out-of-flatness error of the scale grating as well as the form errors of the two-dimensional planar scale grating with an *XY* motorized precision stage [39]. The measurement time could increase in this case and the automation of the *XY* stage, as well as the self-calibration stitching program, is expected to facilitate the calibration process. Future research will include the comparison of the calibrated pitch deviation of the linear scale and the nonlinear component error of the linear optical encoder.

## 5. Conclusions

A self-calibration stitching method based on the Fizeau interferometer, in which the pitch deviation of the linear scale can be evaluated while removing the influence from the reference flat error, has been proposed to evaluate the pitch deviation of a long-range linear scale. The stitching interferometry method has never been applied to evaluate the *X*-directional pitch deviation of the scale grating while eliminating the accumulative second-order effect of the systematic error of the interferometer. Therefore, in this paper, theoretical analysis and simulation have been carried out to develop and test the feasibility of the self-calibration stitching method. Following the theoretical analysis and simulation verification, experiments are conducted by using a long-range linear scale with a length of 105 mm. The pitch deviation of the linear scale can be obtained by directly stitching the pitch deviation from the sub-apertures or evaluated from the firstly stitched order phase outputs, which result to be the same. The PV value of the *X*-directional pitch deviation of the linear scale is evaluated to be 343 nm over its whole area. The pitch deviation evaluation result with three-shot measurement is further compared with the result obtained within one-shot measurement. The high correspondence of the topography in the two assessed pitch deviation maps indicates the capability of the proposed method for long-range linear scale pitch deviation calibration. Meanwhile, the small difference in the averaged *X*-directional cross-sections from the two results also show they have good correspondence with each other. As the first step of the self-calibration stitching of the pitch deviation of the long-range linear scale grating, theoretical analysis has been conducted and primary experiment results have verified the feasibility of the proposed method for the long-range linear scale pitch deviation evaluation. Comprehensive uncertainty analysis of the measurement results, and an extension of the proposed method for the *Z*-directional out-of-flatness measurement of the scale grating as well as the comparison of the evaluated pitch deviation with the optical encoder error will be conducted as future work.

## Figures and Tables

**Table 1:** Error coefficients for each sub-aperture.

	a	b	c
*I_X_*_+1,1_	−2.06	−8.24	−0.59
*I_X_*_+1,3_	1.95	8.37	−0.39
*I_X−_*_1,1_	−2.29	−10.44	−0.66
*I_X−_*_1,3_	2.25	9.98	−0.33
*e_X_*_,1_	28.80	280.04	8.47
*e_X_*_,3_	−38.62	−204.10	−7.02
